# Feasibility and Reproducibility of Isokinetic Dynamometry in Children with Neuromuscular Diseases

**DOI:** 10.3390/jcm13175285

**Published:** 2024-09-06

**Authors:** Danny R. van der Woude, Tim Takken, Thijs Ruyten, Fay-Lynn Asselman, Ruben P. A. van Eijk, W. Ludo van der Pol, Bart Bartels

**Affiliations:** 1 Child Development and Exercise Center, Wilhelmina Children’s Hospital, University Medical Center Utrecht, Lundlaan 6, 3584 EA Utrecht, The Netherlands; dwoude2@umcutrecht.nl (D.R.v.d.W.);; 2 Department of Neurology and Neurosurgery, UMC Utrecht Brain Center, University Medical Center Utrecht, 3584 CX Utrecht, The Netherlands

**Keywords:** isokinetic dynamometry, children, neuromuscular diseases, strength, power

## Abstract

**Background/Objectives:** High-precision measurement tools are needed to measure relevant changes in strength and power in children with neuromuscular diseases. The aim of this study was to determine the feasibility (i), reproducibility (ii), and validity (iii) of isokinetic dynamometry in this population. **Methods:** Isometric and isokinetic knee and elbow flexion and extension were measured twice on the same day. Feasibility was based on completion rate and acceptability. Acceptability was measured with a 100 mm visual analog scale. We measured reproducibility as the intraclass correlation coefficient (ICC-agreement), standard error of measurement (SEM), and smallest detectable change (SDC). We investigated relationships between isometric strength and isokinetic power with Pearson’s correlation coefficient. ROC curves were used to determine the cutoff of isometric strength to conduct isokinetic measurements. **Results:** Fifty children with NMDs participated with completion rates of 78–90% for isometric and 39–75% for isokinetic measurements. Acceptability was high (mean (SD) = 73 (19) mm). The ICCs for all measurements were over 0.9 (95% confidence interval (CI) = 0.932–0.998). The SEM% ranged from 5 to 14% and the SDC% from 14 to 28%. The correlations of strength and power were high (Pearson’s correlation coefficient >0.9 (95% CI: 0.89–0.98)). The isometric strength needed to conduct isokinetic measurements ranged from 11.1 N in elbow flexors to 24.9 N in knee extensors. **Conclusions:** Isokinetic dynamometry is a feasible and reproducible method for measuring isometric strength in children with neuromuscular diseases with moderate weakness to normal strength, and isokinetic measurements are only feasible in knee extension for children with moderate weakness to normal strength. The convergent validity between isometric strength and power at low velocities is high.

## 1. Introduction

Neuromuscular diseases (NMDs) are caused by the dysfunction of the peripheral nervous system at the level of the anterior horn cells, peripheral nerves, neuromuscular junctions, or the muscles [1]. Although the group of NMDs is heterogeneous, progressive muscle weakness is the key characteristic. This makes muscle strength an important outcome measure in studies of disease trajectories, the degree of severity, and treatment efficacy [1,2,3,4]. To measure relevant changes in strength during the course of the disease and in response to treatment, we need high-precision measurement tools that are suitable for children and adults. Commonly used (bedside) strength measurements, such as manual muscle testing (MMT) and handheld dynamometry (HHD), assess isometric strength, i.e., the strength measured in a fixed position. MMT employs the ordinal Medical Research Council (MRC) scale, a 6- to 10-point manual scale, while HHD employs a handheld dynamometer to measure force in a fixed position. Both techniques are easy to use and cheap but have limited inter-rater reliability and sensitivity in stronger patients and larger muscle groups [5,6,7,8,9]. Fixed frame dynamometry or belt stabilized handheld dynamometers address this issue and improve the reliability of isometric measurements but decrease the ease of use. 

The assessment of muscle strength in neuromuscular diseases has long focused on quantifying weakness and documenting the decline in muscle strength. With emerging curative treatments in neuromuscular disease, such as spinal muscular atrophy, Pompe’s disease, and Duchenne muscular dystrophy, there is a need for outcome measures that are reliable and sensitive when measuring stronger patients and can detect meaningful improvements.

Isokinetic dynamometry is considered the “gold standard” in strength testing in orthopedic patients and athletes and has proven to be a reliable tool for measuring both strength and power in healthy adults and children [10,11,12,13,14]. In addition to the possibility of fixed (isometric) dynamometry, IDs can be used to measure dynamic force (i.e., power), expressed in watts [15]. Isokinetic measurements are more comparable to daily life activities compared with isometric measurements [16,17].

A recent review on the reliability of isokinetic dynamometry in NMDs suggests high reliability of strength and power testing in adults with NMDs, although the quality of the evidence was poor, mainly due to small sample sizes [13]. Studies on the feasibility of isokinetic measurements in children have been limited to the strength of the knee extensors [18,19]. 

There have been no studies examining the feasibility or reproducibility of isokinetic dynamometry in children with NMDs. The primary aim of this study was therefore to determine the feasibility and reproducibility of isokinetic dynamometry for measuring isometric strength and isokinetic power in children with NMDs. Second, we aimed to determine the convergent validity of strength and power measured with isokinetic dynamometry and to determine the minimal isometric strength required to conduct isokinetic measurements. 

## 2. Methods

### 2.1. Study Design

We collected data for this observational study with a test–retest design, between January and September 2021. The study procedures were approved by the Medical Ethics Committee of the University Medical Center Utrecht, The Netherlands (20-839/C). Where applicable, the Consensus-Based Standards for the Selection of Health Measurement Instruments (COSMIN) guidelines were followed [20].

### 2.2. Participants

We recruited fifty children with a confirmed diagnosis of an NMD to participate in this study through the pediatric neuromuscular outpatient clinic and the Dutch national SMA registry (www.treatnmd.eu/patientregistries, URL accessed on 28 December 2020) [2]. All children had a medical research council (MRC) MMT score of ≥3 for knee flexors and/or extensors and/or elbow flexors and/or extensors, as determined prior to inclusion. We excluded patients if they could not sit safely in a stable position in the test chair or had a range of motion <90 degrees. The children had to be sufficiently cooperative to perform strength testing with the isokinetic dynamometer (Biodex system 4 pro, Biodex Medical Systems, Shirley, NY, USA). See Table 1 for patient demographic data.

### 2.3. Sample Size

Previous studies on the reliability of isokinetic dynamometry in NMDs and studies on the reliability of isokinetic dynamometry in children report intraclass correlation coefficients (ICCs) ranging from 0.7 to 0.99, with most studies reporting an ICC greater than 0.8 [13,21]. For this study, we used an ICC of ≥0.8 (95% CI ± 0.1) to calculate a sample size of 50 using the formula described by Girandeau and Mary [22]. After collecting data from 50 participants, we calculated ICCs and 95% CIs. We accepted ICCs with a lower limit of the 95% CI of ≥0.8 as sufficient to decide to stop further inclusion of patients. 

### 2.4. Procedures

#### 2.4.1. Equipment

We conducted isometric and isokinetic measurements using a Biodex system pro 4 ID located at the Child Development and Exercise Center in the Wilhelmina Children’s Hospital Utrecht. Biodex systems have proven mechanical reliability in their measurements at velocities up to 500°/s [23,24]. 

#### 2.4.2. Positioning 

We performed measurements on the right side of the body in a seated position, with the backrest in an upright position and 85° hip flexion. The children were fixated using padded straps around their shoulders and waist and at mid-thigh during knee measurements and the upper arm for elbow measurements. For knee measurements, the axis of the dynamometer was aligned with the lateral femoral epicondyle. We placed the force conductor approximately 3.0 cm above the lateral malleoli. The range of motion was set at maximal active flexion and a maximum of 0° extension. During measurements, the arms were kept crossed at the chest. For elbow measurements the dynamometer was rotated 30° away from the upper arm in the sagittal plane. The shoulder was placed in 30° abduction and flexion with the elbow placed on a supporting pad. The axis of the dynamometer was aligned with the lateral epicondyle of the humerus. The handgrip was set at a neutral underarm position. The range of motion was set at a maximal extension of −20° (20° flexion) and maximal 140° of elbow flexion. During measurements, the left hand was placed on the left upper thigh. 

#### 2.4.3. Measurement Protocol

One of two trained pediatric physical therapists (DW and TR) tested the patients twice on the same day for test–retest reliability. Measurements took approximately 30–45 min and were repeated after approximately 2 h of recuperation during which exhausting activities were avoided.

We set the gravity elimination for each measurement in accordance with the Biodex manual [25]. The right arm and right leg were weighed by the system, in maximal knee or elbow extension. The strength and power measurements were adjusted for limb weight by the Biodex system.

For isokinetic knee measurements, we used a velocity of 60°/s based upon the conventional speed in previous studies of isokinetic testing of knee movement in children and patients with NMDs [9,13,19]. For elbow movements, we used 90°/s based on a pilot study with 4 healthy children and 2 children with NMD.

We verbally encouraged children to maximize their effort. We standardized encouragement by using only the direction of movement as a cue in a repetitive, stimulating fashion. At least five practice repetitions were carried out before starting the actual measurement after a 30 s break. We first performed isometric and isokinetic measurements of the knee followed by those of the elbow. If the coefficient of variation (COV) was >15% for knee measurements or >20% for elbow measurements, the test was repeated once. After the final measurement, we asked the patients to rate their willingness to do the assessments again in the future on a 100 mm visual analog scale. We used this score to determine the acceptability.

Isometric measurements started with a practice test of at least five consecutive extension and flexion attempts at a 90-degree fixed position, so that the patient could become acquainted with the measurement technique. The actual test consisted of three consecutive cycles of maximal efforts in flexion and extension directions in a fixed position, with a duration of five seconds per contraction and a five-second interval between directions. 

Isokinetic measurements started with a practice test of at least five movements over the full range of motion so that the patient could become acquainted with the speed and movement. Following the practice movements, five consecutive knee flexion and extension movements over the full, preset, range of motion were made at maximum strength, starting with extension movement. 

### 2.5. Data Analysis

We used IBM SPSS Statistics for Mac, version 25 (IBM Corp. 2017), to conduct all statistical analysis. We performed statistical analysis for each type of measurement and direction (i.e., isometric or isokinetic, knee or elbow, flexion or extension). Due to the skewed distribution of muscle strength, we used log_10_ transformations [26]. Bland–Altman plots were used to check for heteroscedasticity. We created Bland–Altman plots by plotting the difference between test and retest measurements (test minus retest) against the mean of the test and retest measurements of each patient [27]. We performed paired *t*-tests to detect systematic differences between test and retest that could indicate potential bias (e.g., due to fatigue or a learning effect).

#### 2.5.1. Feasibility

To determine the *acceptability*, the mean score and standard deviation (SD) were calculated for the VAS score given following the question, “Would you mind doing this measurement again?” We accepted a mean score of >60 mm as acceptable and reported the number and percentage of patients scoring >60 mm.

The *completion rate* was expressed as the percentage of patients able to conduct isometric and/or isokinetic measurements under the conditions of ability to achieve the preset velocity and quality of the measurement, calculated with the formula percentage = $\frac{n^{succes}}{n^{Total}}\times 100$. 

The quality of isokinetic dynamometry is based on the variation of exerted torque within one measurement, which consist out of multiple repetitions, expressed as the coefficients of variation (COVs). High COVs are clinically regarded as non-valid measurements and not used. Therefore, only measurements with acceptable quality are used to determine reproducibility. We included patients with a COV of ≤15% for knee measurements and a COV of ≤20% for elbow measurements. A COV ≤15% was considered acceptable for measurements of large muscle groups, and a COV of ≤20% was considered acceptable for smaller muscle groups [28].

A score of ≥70% was qualified as feasible. We calculated the completion rate at the group level and of the weaker (MRC 3) and stronger groups (MRC ≥ 4). Measurements with sufficient quality were included in the reproducibility part of the study (Supplementary Materials Figure S1).

#### 2.5.2. Reproducibility

Reproducibility can be divided into reliability and measurement error [29]. Reliability, expressed using the intraclass correlation coefficient (ICC), refers to the test’s ability to distinguish patients from each other despite measurement errors [30,31]. The ICC is the proportion of the total variance in the measurement that can be attributed to between-patient variability [32]. Higher ICCs reflect less within-patient variability (random error) and more consistent measurements within an individual. Measurement error refers to changes in the measurements due to systematic and random errors [32]. We also calculated the standard error of measurement (SEM) and the smallest detectable change (SDC), expressed as a percentage. The SDC was calculated as $\mathrm{SEM}\times 1.96\times \sqrt{2}\;$; changes surpassing the limits of the SDC indicated true change [33]. 

#### 2.5.3. Reliability

We calculated ICC_agreement_ using a two-way analysis of variance (ANOVA, 2-way random-effects model, absolute agreement, 95% confidence interval, single measures) on log_10_ transformed data [29,34]. We calculated the ICCs for knee flexion, knee extension, elbow flexion, and elbow extension, at the outcome measures peak torque for isometric measurements and peak torque and average power for isokinetic measurements.

#### 2.5.4. Measurement Error

We calculated the SEM_agreement_ and SDC on log_10_ transformed data and back-transformed to percentages, SEM% and SDC% [26,29,30,33].

#### 2.5.5. Validity

We assessed the correlation of the isometric strength (peak torque) and power (watts) from the first measurements for related measurements (i.e., knee flexion, knee extension, elbow flexion and elbow extension) using Pearson’s correlation coefficient, “r”.

#### 2.5.6. Minimal Isometric Strength to Conduct Isokinetic Measurements

We used receiver operating characteristics (ROC) curves to determine the optimal cutoff of isometric strength to conduct isokinetic measurements. First, we selected all children who were able to conduct isometric measurements. Second, we labeled the isokinetic measurements as “executed” (able to conduct isokinetic measurements) or “not executed” (unable to conduct isokinetic measurements), irrespective of the COV% of the isokinetic measurements.

## 3. Results

Table 1 shows the patient characteristics of all 50 included patients. 

### 3.1. Feasibility 

Acceptability was sufficient with a mean (SD) score of 73 (19) mm. Forty-one patients (84%) scored ≥60 mm. Eight patients (16%) scored <60 mm, of whom six (12%) scored between 51 and 57 mm and two (4%) scored 5 mm and 21 mm. The acceptability scores of one participant were not available.

The completion rate (Table 2) at the group level was acceptable for isometric measurements for knee and elbow movements with a completion rate of 78–90%. When divided into the weaker (MRC 3) and stronger (MRC ≥ 4) groups, isometric measurements were only feasible in the stronger group with completion rates of 40% to 64% and 84% to 97%, respectively. For isokinetic measurements, the completion rate at the group level was only acceptable for knee extension (75%). When divided into the weaker (MRC 3) and stronger (MRC ≥ 4) groups, isokinetic measurements were not feasible in the weaker group and only feasible for knee extension (88%) and somewhat feasible for knee flexion (66%) in the stronger group.

The reasons for unsuccessful measurement were inability to move the force conductor or inability to exert enough force to start the measurement (77%) and low quality of measurement (23%) (Supplementary Materials Table S1). 

### 3.2. Reproducibility

**Figure 1:**
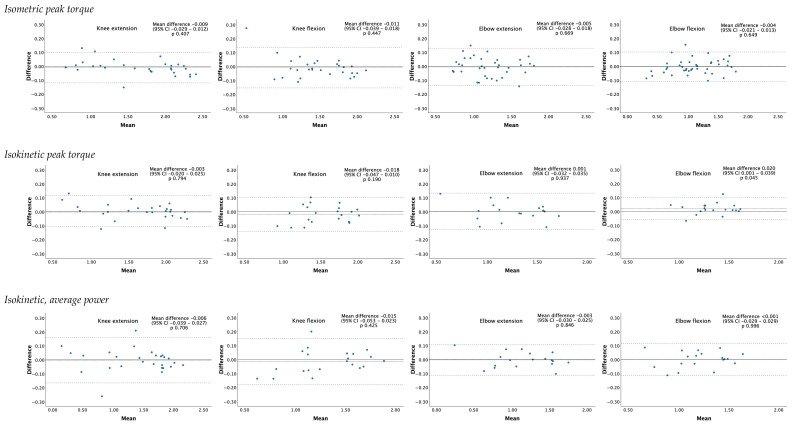
Bland–Altman plots of strength and power measurements. The x-axis shows the mean score (test + retest/2), and the y-axis shows the difference between test and retest (retest–test). The horizontal straight line, at zero difference, represents no mean change between test and retest. The middle dotted line represents the mean difference between test and retest. The dotted outer lines represent the 95% limits of agreement (mean ± 1.96 SD). Each black dot represents one patient; the closer the dots are to zero, the higher the agreement between test and retest, indicating lower measurement error.

The Bland–Altman plots showed no heteroscedasticity. Outliers (measurements outside the 95% CI) are projected in Figure 1 as dots surpassing the outer dotted lines (Figure 1). *t*-tests only showed a significant difference with mean difference 0.02 (95% CI 0.001 to 0.039, *p* = 0.045) between test and retest for peak torque in isokinetic elbow flexion measurements (Table 3).

#### 3.2.1. Reliability

Reliability was excellent for all measurements (ICC lower limit of 95% CI > 0.9) (Table 3). 

#### 3.2.2. Measurement Error

The measurement error was 9–12% for isometric measurements and 7–14% for isokinetic measurements. The SDC% ranged from 24 to 33% in isometric measurements and from 20 to 38% in isokinetic measurements.

### 3.3. Convergent Validity

**Figure 2:**
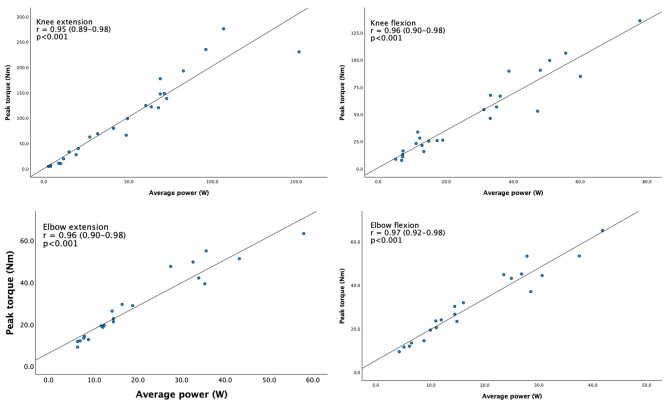
Correlation plots between average power (isokinetic) on the *x*-axis and peak torque (isometric) on the *y*-axis. Each dot represents one patient. n = 25 for knee flexion and knee extension, and n = 21 for elbow flexion and elbow extension. Pearson’s r (95% CI).

We observed linearity for all related measurements (Figure 2). Convergent validity between the isometric peak torque and the isokinetic (average) power was Pearson’s r = 0.95 (95% CI 0.89 to 0.98) for knee extension, r = 0.96 (95% CI 0.90 to 0.98) for knee flexion, r = 0.96 (95% CI 0.90 to 0.98) for elbow extension, and r = 0.97 (95% CI 0.92 to 0.98) for elbow flexion.

### 3.4. Minimal Isometric Strength to Conduct Isokinetic Measurements

The optimal cutoff for the minimal isometric strength needed for isokinetic measurements ranged from 11.1 Nm in elbow flexion to 24.9 Nm in knee extension (Table 4).

## 4. Discussion

This study aimed to determine the feasibility and reliability of isokinetic dynamometry in children with NMDs and the convergent validity between isometric strength and power. Isokinetic dynamometry was a feasible and reproducible method for measuring isometric strength in children with an NMD who had moderate weakness to normal strength (MRC ≥ 4). The measurement of power using isokinetic dynamometry was only feasible for knee movements in children with MRC ≥ 4. The reproducibility of isokinetic dynamometry was excellent. Isometric strength and isokinetic power at low velocity showed strong convergent validity, indicating they both related to the same construct of muscle function.

Our results on feasibility were in line with previous studies on reliability and feasibility of isokinetic dynamometry in adult populations with NMDs. Seventy-seven percent of unsuccessful isokinetic measurements were due to children being unable to exert sufficient force to start the measurement or to move the force conductor at the preset velocity. Previous studies on isokinetic dynamometry in NMD also report patients being unable to perform the measurements [35,36,37], while studies on healthy children do not report these limitations [18,19], indicating that a lack of strength limits the feasibility of, particularly, isokinetic measurements. 

To improve the feasibility of isokinetic dynamometry in weaker children, the use of a passive mode might be an alternative. Studies on isokinetic dynamometry set up in continuous passive mode, where the force conductor moves on its own, show high reliability in patients with MRC 2 and 3 [38,39]. This type of isokinetic measurement in children with NMDs might reveal a possible way of measuring strength and power in patients who are unable to exert enough force to start the measurement or to measure force or patients unable to move the force conductor at the preset speed.

The reproducibility of isokinetic dynamometry was, on the whole, in line with previous studies on adults with NMDs [13]. Compared with healthy children, ICCs of knee measurements were higher, possibly explained by differences in velocity, number of repetitions, patient placing, and encouragement [19,40]. The SEM% and SDC% varied from being comparable to being slightly lower compared with other studies on NMDs and healthy children [13,21,40].

Although surpassing the limits of the SDC% indicates true change, these limits do not necessarily indicate changes that are clinically relevant. The minimal clinically important difference (MCID) refers to the difference considered important or beneficial to patients; therefore, if MCID < SDC, the measurement error may conceal clinically important changes in patients [41,42]. Ideally, MCIDs are determined using anchor-based approaches [32]. Currently, there are no studies on anchor-based approaches to determine the MCID of strength or power in NMDs. Therefore, distribution-based MCIDs require interpretation with caution as they are not based on what patients consider to be important changes, but are calculated and, therefore, might lack clinical relevance [32]. Our study falls just short of the (distribution-based) MCIDs reported on isometric knee strength in myotonic dystrophy [35] and Duchenne muscular dystrophy [43]. This indicates that isometric measurements of strength in myotonic dystrophy or Duchenne muscular dystrophy might conceal clinically relevant changes, albeit small. More research is needed to determine proper (anchor-based) MCIDs for strength and/or power in children with NMDs. In the meantime, the use of functional scales and patient-reported outcomes in clinical practice might contribute to a broader understanding of the correlation between relevant changes and improvements in strength. 

This study focused on the relationship between strength and power generated at relatively low velocities. Studies on correlations of strength and power at higher velocities tend to show lower correlation [44], possibly due to differences in muscle fiber type recruitment within the muscle [45]. Relatively higher amounts of fast twitch fibers will likely lead to higher power at higher velocities. Further research is needed to determine the correlation between strength and power at higher velocities in children with NMDs

### 4.1. Study Strengths and Limitations

The large number of children with SMA included in this study may represent a source of bias. However, there was a wide variation of muscle strength between patients, and feasibility was demonstrated at the group and MRC levels. SMA patients did not show lower ICCs or larger variety in measurement error compared with the rest of the group of children with NMDs. Therefore, the large population of SMA patients did not affect feasibility or reproducibility. 

The cutoff values for isokinetic measurement are useful estimates and can be used as a guideline in clinical practice. When applying these limits in research, valuable data might be missed or neglected, with successful measurements falling below the cutoff for all movements. Future research focused on the relation of HHD measurements and isometric measurements at an isokinetic dynamometer could be useful and relevant. The use of cutoff values measured with HHD to determine the feasibility of isokinetic measurements saves time and effort and improves the use of isokinetic dynamometry in clinical care and research. 

### 4.2. Implications

This study provides feasibility and reproducibility data on isokinetic dynamometry in children with NMDs. Applying standardized protocols for positioning and encouragement have likely contributed to higher reliability and smaller measurement error. The outcomes of this study support its use in the longitudinal assessments of strength and power in children with NMDs and relatively spared muscle strength, especially when small changes are relevant. When participants have less muscle strength (MRC < 4), the benefit of measuring power is less feasible, and the use of (stabilized) HHD might form an equal possibility to measure isometric strength.

## 5. Conclusions

This study shows that isometric measurements are feasible and reproducible in children with NMDs with moderate weakness to normal strength and that the use of isokinetic dynamometry to measure power is only feasible in knee movements in children with preserved muscle strength (MRC ≥ 4). Strength and power measurements show high correlations when measured at relatively low velocities, indicating both measurements rely on the same force-developing properties of the muscle. More research is needed to examine ways of measuring isokinetic strength and power in weaker children with NMDs and to determine the correlations of strength and power at higher velocities.

## Figures and Tables

**Table 1:** Demographic data.

Age, mean (SD), y	12.1 (3.9)
Body mass, mean (SD), kg	44.9 (18.0)
Height, mean (SD), cm	149.8 (21.7)
Sex, female, n (%)	22 (44%)
Ambulatory status, ambulatory, n (%)	34 (68%)
MRC knee extensors, n	36
MRC 3, n (%)	11 (31%)
MRC ≥ 4, n (%)	25 (69%)
MRC knee flexors, n	37
MRC 3, n (%)	5 (14%)
MRC ≥ 4, n (%)	32 (86%)
MRC elbow extensors, n	40
MRC 3, n (%)	5 (12.5%)
MRC ≥ 4, n (%)	35 (87.5%)
MRC elbow flexors, n	49
MRC 3, n (%)	14 (29%)
MRC ≥ 4, n (%)	35 (71%)
Diagnosis, n (%)	
Axonal motor neuropathy	1 (2%)
Axonal polyneuropathy	1 (2%)
Becker muscular dystrophy	3 (6%)
Carey Fineman Ziter syndrome	2 (4%)
Central core myopathy	1 (2%)
Duchenne muscular dystrophy	1 (2%)
Hereditary motor and sensory neuropathy	7 (14%)
Myotonic dystrophy	1 (2%)
Myasthenia gravis	2 (4%)
Myotonica congenita (type Becker)	2 (4%)
Nemaline myopathy	1 (2%)
Silver syndrome	1 (2%)
Spinal muscular atrophy	27 (54%)
1c	1 (2%)
2a	7 (14%)
2b	1 (2%)
3a	17 (34%)
3b	1 (2%)

MRC (Medical Research Council) scale.

**Table 2:** Completion rate.

Measurement	MRC 3	MRC 4 and Higher	Total
Isometric			
Knee extension	7/11 (64%)	22/25 (88%)	29/36 (81%)
Knee flexion	2/5 (40%)	27/32 (84%)	29/37 (78%)
Elbow extension	2/5 (40%)	34/35 (97%)	36/40 (90%)
Elbow flexion	8/14 (57%)	33/35 (94%)	41/49 (84%)
Isokinetic			
Knee extension	5/11 (46%)	22/25 (88%)	27/36 (75%)
Knee flexion	0/5 (0%)	21/32 (66%)	21/37 (57%)
Elbow extension	1/5 (20%)	17/35 (49%)	18/40 (45%)
Elbow flexion	0/14 (0%)	19/35 (54%)	19/49 (39%)

**Table 3:** Test–retest reliability, standard error of measurement, and smallest detectable change.

Measurement	Test, Median (IQR)	Retest, Median (IQR)	*p*-Value ^a^	ICC ^b^	95% CI for ICC	SEM% ^c^	SDC% ^c^
*Isometric*							
Knee extension							
Peak torque (Nm)	65.9 (12.2–142.8)	61.0 (13.3–124.8)	0.407	0.995	0.990–0.998	9%	25%
Knee flexion							
Peak torque (Nm)	26.2 (16.1–67.1)	27.5 (14.8–63.4)	0.447	0.983	0.964–0.992	12%	33%
Elbow extension							
Peak torque (Nm)	14.7 (7.8–28.3)	13.6 (9.6–25.2)	0.669	0.977	0.955–0.988	11%	31%
Elbow flexion							
Peak torque (Nm)	11.5 (7.2–25.3)	11.4 (7.5–24.8)	0.649	0.991	0.983–0.995	9%	24%
*Isokinetic*							
Knee extension							
Peak torque (Nm)	62.8 (15.8–111.0)	65.1 (16.6–114.2)	0.794	0.988	0.974–0.994	9%	25%
Average power (W)	40.4 (9.2–68.8)	44.9 (9.0–63.3)	0.706	0.990	0.978–0.995	13%	37%
Knee flexion							
Peak torque (Nm)	27.6 (20.6–72.1)	27.0 (20.0–65.5)	0.190	0.981	0.955–0.992	10%	29%
Average power (W)	18.9 (11.7–43.0)	18.1 (12.4–40.4)	0.425	0.972	0.934–0.989	14%	38%
Elbow extension							
Peak torque (Nm)	19.9 (12.0–33.4)	18.0 (12.1–34.2)	0.937	0.979	0.946–0.992	11%	29%
Average power (W)	14.4 (8.6–33.3)	12.4 (8.6–33.2)	0.846	0.991	0.977–0.997	9%	25%
Elbow flexion							
Peak torque (Nm)	18.1 (12.5–31.4)	19.2 (11.3–33.4)	0.045	0.977	0.932–0.991	7%	20%
Average power (W)	14.4 (8.3–27.3)	15.3 (7.4–28.1)	0.996	0.978	0.944–0.992	9%	26%

^a^ *p*-value calculated with log-transformed data; ^b^ ICC calculated with log-transformed data; ^c^ No absolute value, only ratio calculated with log-transformed data. IQR (interquartile range), ICC (intraclass correlation coefficient), CI (confidence interval), SEM (standard error of measurement), SDC (smallest detectable change), Nm (Newton meter), W (watt).

**Table 4:** Isometric strength needed to conduct isokinetic measurements.

Measurement	Isokinetic Measurement Executed (n)	Isokinetic Measurement Not Executed (n)	Area Under the Curve (95% CI)	Cutoff (Nm)	Successful Measurements Below Cutoff (n)
Knee extension (n = 30)	25	5	0.86 (0.73–1)	24.9	7
Knee flexion (n = 31)	26	5	0.81 (0.62–0.99)	20.3	7
Elbow extension (n = 36)	22	14	0.94 (0.86–1.0)	17.6	7
Elbow flexion (n = 46)	23	23	0.90 (0.80–1.0)	11.1	4

## Data Availability

The data generated in this study are available upon reasonable request.

## Supplementary Materials

The following supporting information can be downloaded at: https://www.mdpi.com/article/10.3390/jcm13175285/s1. Figure S1. Flowchart inclusion. Table S1. Reasons for unsuccessful measurements.
