# Supplementary material for: Feasibility and Reproducibility of Isokinetic Dynamometry in Children with Neuromuscular Diseases
Source: J Clin Med. 2024 Sep 6;13(17):5285. doi: 10.3390/jcm13175285 (PMC11396260; doi:10.3390/jcm13175285)
Supplement: Supplementary file 1 [file jcm-13-05285-s001.zip › jcm-3134456-supplementary.pdf]

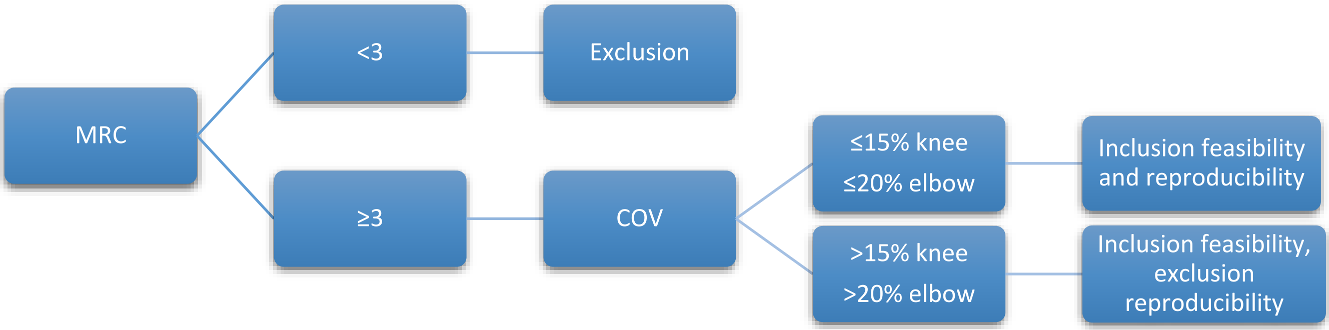

Figure S1. Flowchart inclusion.

Table S1. Reasons for unsuccessful measurements.

**Measurement**	Reason for unsuccessful measurement	
	Unable to move force conductor/start measurement	COV >15/20%
Isometric		
Knee extension	6/7 (86%)	1/7 (14%)
Knee flexion	6/8 (75%)	2/8 (25%)
Elbow extension	3/4 (75%)	1/4 (25%)
Elbow flexion	6/8 (75%)	2/8 (25%)
Isokinetic		
Knee extension	9/9 (100%)	0/9 (0%)
Knee flexion	10/16 (63%)	6/16 (37%)
Elbow extension	16/22 (73%)	6/22 (27%)
Elbow flexion	24/30 (80%)	6/30 (20%)
Total	80/104 (77%)	24/104 (23%)
